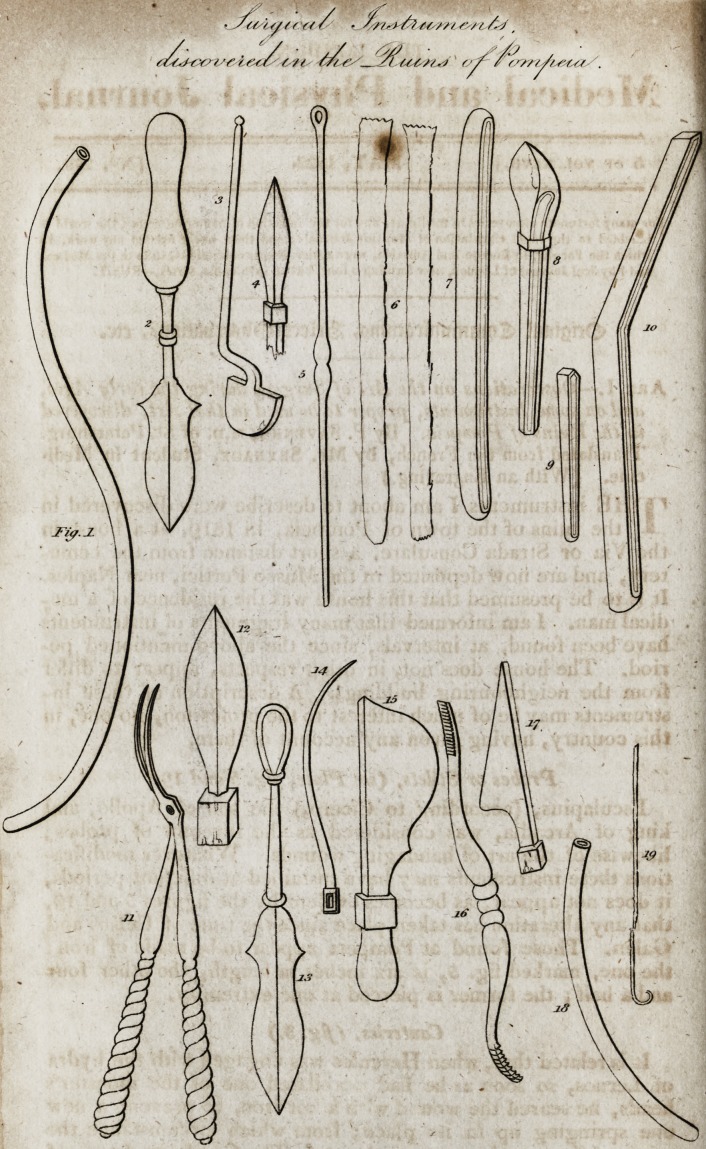# Observations on the Art of Surgery during the Early Ages, and on Some Instruments, Proper to Be Used in That Art, Discovered in the Ruins of Pompeia

**Published:** 1822-05

**Authors:** P. Savenko

**Affiliations:** St. Petersburg.; Student in Medicine.


THE LONDON
### Medical and Physical Journal.

5 OF VOL. XLVII.] MAY, 1822. [No. 279.

For many fortunate discoveries in medicine, and for the detection of numerous errors, the world is indebted to the rapid circulation of Monthly Journals; and there never existed any work, to which the Faculty, in Europe and America, were under deeper obligations, than to the Medical and Physical Journal of London, now forming a long, but an invaluable, series.—RUSH.

### Original Communications, Select Observations, etc.

**Art. I.**—*Observations on the Art of Surgery during the early Ages, and on some Instruments, proper to be used in that Art, discovered in the Ruins of Pompeia.* By P. Savenko, m.d. of St. Petersburg. Translated from the French, by Mr. Seynage, Student in Medicine. (With an Engraving.)

THE instruments I am about to describe were discovered in the ruins of the town of Pompeia, in 1819, at a house in the Via or Strada Consulare, a short distance from the cemetery, and are now deposited in the Muséo Portici, near Naples. It is to be presumed that this house was the residence of a medical man. I am informed that many fragments of instruments have been found, at intervals, since the above-mentioned period. The house does not, in other respects, appear to differ from the neighbouring buildings. A description of these instruments may be of much interest to the profession, no one, in this country, having given any account of them.

*Probes or Stilets, (see Plate, fig. 5 and 19.)*

Esculapius, (according to Cicero,) the son of Apollo, and king of Arcadia, was considered as the inventor of probes; likewise of the art of bandaging wounds. Whatever modifications these instruments may have sustained at different periods, it does not appear, as becomes evident by the figures 5 and 19, that any alteration has taken place since the time of Celsus and Galen. Those found at Pompeia appear to be made of iron: the one, marked fig. 5, is six inches in length, the other four and a half; the former is pierced at one extremity.

*Cauteries, (fig. 3.)*

It is related that, when Hercules was engaged with the hydra of Lernos, so soon as he had decollated one of the monster's heads, he seared the wound with a hot iron, to prevent a new one springing up in its place; from which circumstance the origin of the actual cautery is derived. The Greeks made use of it in the early ages, and appear to have borrowed the custom from the Egyptians or Ethiopians; and the Lybians, inhabitants of Africa, who made much use of it, especially those who led a pastoral life, were in the habit of burning the veins of the upper part of the head and temples of their children, when they had attained the age of four years, with greasy wool, as a preventive to many incommodities. (*Herodotus*, lib. iv.) The application of fire was equally common amongst the Scythians.

Hippocrates affirms that diseases, which are not cured by medicines, are removable by iron: should that fail, fire is to be employed; if the malady still continues, it is incurable. This great man was accustomed to apply the actual cautery in pains of the head, which did not yield to common remedies.

Archagatus, the Peloponesian, (and the first physician who settled in Rome,) acquired, at first, much distinction; but ultimately became very unpopular, and was commonly called the executioner, on account of his partiality to manual operations, and the frequent application of the actual cautery.

The celebrated surgeon, A. Cornelius Celsus, appeared at a later period, and was an acknowledged partizan to the use of the cautery.

About the same time, under the reign of Claudius, the application of fire became of considerable service in the treatment of the mentagra, and was used successfully by the Egyptian physicians, who had been sent for on the appearance of this disease.

It will be seen that the irons used by the ancients are nearly similar to those of the present day. That represented in fig. 3 is of iron, and is four inches in length; the extremity, used for cauterizing, has the appearance of a full horse-shoe, and a ball at the other end renders it probable that it had no other handle.

*Instruments for Bleeding, (fig. 4 and 12.)*

We possess no certain information on the origin of phlebotomy. The first example we know of occurred at the siege of Troy. It is related that Podalirus, (one of the most skilful surgeons amongst the Greeks,) was, on returning from Troy, cast upon the coast of Caria, where he cured Syrna, daughter of Damætus, king of that country, by bleeding her in both arms. Some authors place the origin at a more remote period: among others, Pliny affirms that this custom was taught to men by the hippopotamus, who, on the banks of the Nile, rubs his legs against the reeds until a plentiful discharge of blood has taken place. The Egyptians contend that they were the first who knew of this operation. It was a custom with the Scythians, when they were indisposed, to divide a vein behind each ear, and permit it to bleed till fainting took place; and it is known that the negroes, when they imagine they have a superabundance of blood, are in the habit of stabbing themselves in any part of the body with a knife, to ease themselves of a sufficient quantity of that fluid. At Congo, they use a small sharpened shell; at Tonquin, the bone of a fish. When the island of Taiti was discovered, the inhabitants were well acquainted with blood-letting: the operation was performed by their *tavera*, or druids, by striking upon the skull with a sharp piece of wood, and thus opening one of the sagittal veins. The head was bound up after a quantity of blood had been lost.

In the time of Hippocrates, the veins of the forehead and tongue were frequently opened; but we are unacquainted with the instruments they made use of. At a later period, it is said that Celsus used a scalpel, which, in the opinion of some, is shaped like our modern lancet: it is therefore conjectured that the instruments, figs. 4 and 12, were used to bleed with. It is to be remarked, also, that these, as well as many others used by the ancients, are made of copper, and that their hardness is but little inferior to steel.

The instrument, fig. 4, is two inches and a half in length, and fig. 12 is three inches, shaped like a spear, the greatest width being in the middle, and are both coated with a green oxide.

*Cutting Instruments, (fig.* 15 *and* 17.)

We are as ignorant of the origin of knives among the ancients as of their lancets: all we know is, that, when Hippocrates flourished, lithotomy was an operation often performed, as well as that of trepanning, empyema, &c.; and water was evacuated in ascites and anasarca, by tapping or scarifications; abscesses were punctured, as well as glands when in a state of suppuration. Asclepiades, of Bithynia, was the first who advised, or, as some say, practised, the operation of bronchotomy, to prevent the fatal results of severe angina. Celsus describes the method of amputating limbs; he recommends that the muscles should be divided upon the bone, and to saw the latter. (Lib. vii. cap. iv. sect. iii.) He was acquainted with all the operations that had been practised before him, and his name is attached to a treatise on Lithotomy. It is easily conceived that many different kinds of knives were necessary to render these operations practicable; but we have not any description of most of them, and we are unacquainted with the respective use of those that are known.

Fig. 15th represents a blade, two inches and a half in length and very wide, the back of which is straight and thick, and the edge convex. It appears to be broken near the handle, which is very short. The blade of the scalpel, fig. 17th, is two inches in length, the greatest breadth being one inch; its shape an elongated triangle; a straight back terminating in a sharp point, and gradually widening near the handle. This instrument is of copper; and the one, fig. 15, much altered by rust, appears to be of the same metal.

*Instrument for extracting Teeth, (fig.* 11.)

It is not known whether the extraction of teeth was practised in the time of Hippocrates. It is probable, by what we read in Celsus, that, even in his time, loose teeth only were extracted, as the forceps then used were very imperfect. This induces us to consider the instrument, marked fig. 11, to be one of them; the two fangs of which are of iron, curved, and indented. The handle is two inches in length, the fangs not quite so long.

*Instruments for Trepanning, (fig.* 16.)

Hippocrates is the first known to have noticed this operation, he having mentioned it in his book on Wounds of the Head, in a manner which leaves no doubt of his having performed it; he even describes the instruments he made use of. Since his time this operation has often been performed. Fig. 16 represents an elevator, entirely similar to those used in the present day.

*Pincers, (figs.* 6, 7, 8, 9, *and* 10.)

From the most distant period, hooks, pincers, and other instruments, were used to remove extraneous substances from wounds. No doubt they were not numerous, and have been multiplied gradually as the science has improved. Those represented in figs. 6, 7, 8, 9, and 10, are of this kind; perhaps they were used for purposes with which we are unacquainted. Figs. 6 and 7 are five inches in length; fig. 8 is three and a half in length, and pointed at the extremity; fig. 9 is the same kind as those used by our oculists to extract the eye-lashes; fig. 10 is six inches in length, and appears adapted to extract any thing deep seated.

*Sounds or Catheters, (figs.* 1 *and* 18.)

The catheter, fig. 1, is nine inches in length; its diameter equal in every part; its thickness cannot be determined, on account of the quantity of rust, which renders it scarcely pervious: it appears to be of iron, though Celsus calls them *fistulæ æneæ*. It is a curious circumstance that its shape, which resembles an italic *s*, considered so useful when required to remain any length of time in the bladder, was supposed to be the invention of J. Louis Petit some years back. Fig. 18 is probably a female catheter, and is four inches in length.

*Fig.* 14.

A needle resembling our seton needles. Query, was it used for that purpose? It is of iron, and is three inches in length.

*Figs. 2 and 13,*

Are spatulas, the shape of those we often meet with; they are of iron, if we may judge by the rust which covers them. Fig. 2 is a double one; 13 has a handle shaped like a spoon.

## Figures and Tables

**Fig. 1 2 3 4 5 6 7 8 9 10 11 12 13 14 15 16 17 18 19 f1:**